# Substance Use Disorders in Patients With Parkinson’s Disease and Adverse Hospitalization Outcomes: A National Inpatient Study

**DOI:** 10.7759/cureus.16033

**Published:** 2021-06-29

**Authors:** Jaskaranpreet Kaur, Ramneek K Sandhu, Khadija T Kubra, Johanna S Canenguez Benitez, Henry K Onyeaka, Sabiha Akter, Ozge C Amuk Williams

**Affiliations:** 1 Internal Medicine, Dayanand Medical College and Hospital, Punjab, IND; 2 Internal Medicine, Shri Guru Ram Das Institute of Medical Sciences and Research, Amritsar, IND; 3 Internal Medicine, Bangladesh Medical College and Hospital, Dhaka, BGD; 4 Internal Medicine, Larkin Community Hospital, Houston, USA; 5 Psychiatry, Harvard School of Medicine, Boston, USA; 6 Psychiatry, Bergen New Bridge Medical Center, Paramus, USA; 7 Psychiatry, Koç University School of Medicine, Istanbul, TUR

**Keywords:** parkinson’s disease, barbiturate use, opioid use, stimulants use, hospitalization outcomes

## Abstract

Objectives

To understand the demographic pattern of substance use disorders (SUD) in Parkinson’s disease (PD) inpatients and to evaluate the impact of SUD on hospitalization outcomes including the severity of illness, length of stay (LOS), total charges, and disposition to nursing facilities.

Methods

We used the nationwide inpatient sample and identified adult patients (age, ≥40 years) with PD as a primary diagnosis and comorbid SUD (N = 959) and grouped by co-diagnosis of alcohol (N = 789), cannabis (N = 46), opioid (N = 30), stimulants (N = 54) and barbiturate (N = 40) use disorders. We used a binomial logistic regression model to evaluate the odds ratio (OR) for major loss of functioning and disposition to nursing facilities in PD inpatients. All regression models were adjusted for demographics, including age, sex, race, and median household income.

Results

Alcohol, opioid, and stimulant use disorders were prevalent in old-age adults (60-79 years), males, and whites, but cannabis use was prevalent in middle-aged adults (40-59 years), and barbiturate use among older-age (>80 years). The severity of illness is statistically higher in PD inpatients with comorbid opioid and barbiturate use disorders with major loss of body functioning, closely seconded by alcohol and stimulant use disorder cohorts (27.6% and 25.9%, respectively). Disease severity and loss of body functioning increase with advancing age (>80 years adults, OR 5.8, 95%CI 5.32-6.37), and in blacks (OR 1.7, 95%CI 1.56-1.81), and those with opioid use disorder (OR 3.8, 95%CI 1.96-7.35). PD inpatients with barbiturate use disorder had a higher LOS and charges by 17.4 days and $68,922, and six-fold increased likelihood (95%CI 2.33-15.67) for disposition to nursing facilities.

Conclusions

SUD is prevalent among PD patients and is associated with more severe illnesses with body loss functioning and prolonged care. A multidisciplinary care model including collaborative neuropsychiatric and addiction management is required to manage SUD among PD patients to lessen disease severity, slow down the disease progression and potentially save medical costs.

## Introduction

Parkinson’s disease (PD) is the second most prevalent multisystem synucleinopathic neurodegenerative disorder in North America [1]. The national center for health statistics center estimates the incidence of PD to rise to nearly one million by 2020 and 1.25 million by 2030 in the United States (US) owing to the rapidly growing aging population [2]. PD is characterized by a constellation of motor and non-motor symptoms owing to the degeneracy and depletion of dopaminergic neuronal in the cardio and nephritic vasculature, central (CNS), peripheral (PNS), and enteric nervous systems [3,4].

PD is a disease with no cure or prevention that often begins with minimal prodromal symptoms but ultimately manifests into a myriad of disabling symptomatology progressively increasing in severity with advancing age [1]. The dopamine receptor 3 (d3), localized within the limbic area, is the primary receptor involved in the compulsive perseverance associated with reward and reinforcement loops that mediate drug-seeking behaviors amongst substance use disorder patients [4]. Furthermore, recent studies implicate the dopamine receptor 4 (d4) to be involved in the relapse to stimulant abuse, whilst dopamine receptors 1 and 2 (d1 and d2, respectively) predominantly work within a highly coordinated synaptic network encompassing the prefrontal cortex, hippocampus, and the amygdala [3].

Given the chronicity, progressive clinical deterioration, and gross morbidity of the disease, patients were believed to often resort to absolving their non-motor symptoms by turning to illicit substances that often lead to comorbid substance use disorders (SUD). Novel research findings suggest early-onset illicit substance use and chronic use of stimulants may potentiate the early-onset parkinsonism, eventually leading to the disease [5]. In this study, we seek to look at the five most abused classes of substances among PD patients, including barbiturate, stimulants (cocaine and amphetamine), opioids, and cannabis. We used the nationwide inpatient sample (NIS) to firstly understand the demographic pattern of SUD in PD inpatients, and next to evaluate the odds of association between severity of illness and disposition to nursing facilities in PD inpatients and across the spectrum of SUD.

## Materials and methods

Data source and sample

A retrospective study was conducted using the healthcare cost and utilization project's (HCUP) NIS data for the year 2014. The NIS database is considered to determine patterns in demographics and hospitalization outcomes. It is the largest inpatient database which covers 4,411 hospitals from 44 states in the United States. In order to protect the privacy of individual patients, physicians, and hospitals, the state and hospital identifiers were de-identified [6]. So, we were not required the institutional review board's permission to conduct a study on publicly available de-identified data.

We extracted a sample of 62,784 adult patients (age, ≥40 years) with a principal diagnosis of PD based on the International Classification of Diseases, ninth revision (ICD-9) diagnosis code of 332. An analysis of the sample data extrapolated from the NIS found that 959 (1.25%) patients had comorbid diagnoses of SUD, and was grouped by comorbid alcohol use disorder (N = 789), cannabis use disorder (N = 46), opioid use disorder (N = 30), stimulant use disorders (N = 54) and barbiturate use disorder (N = 40).

Variables of interest

The demographic variables included in this study were age, sex, race, and median household income. In order to measure the differences in hospital outcomes in PD patients by the presence of comorbid SUD, included the following variables: severity of illness, length of stay (LOS), total charges, and disposition to a skilled or intermediate nursing facility (SNF/INF). In the NIS, all patient refined DRGs (APR-DRGs) are assigned using software developed by 3m health information systems to classify the severity of illness into minor, moderate, and major loss of body function. LOS is the number of nights the patient remained in the hospital for the principal diagnosis of PD, and total charges during PD hospitalization do not include professional fees and non-covered charges [7].

Statistical analyses

We used descriptive statistics and linear-by-linear association tests for categorical data and independent sample t-test for the continuous data to measure the differences in demographics and hospital outcomes across the spectrum of SUD. We used the discharge weight variable, which is given in the NIS, to attain a national representation of the sample population. We used a binomial logistic regression model to evaluate the odds ratio (OR) for major loss of functioning and disposition to SNF/INF in PD inpatients with the spectrum of SUD. All regression models were adjusted for demographics including age, sex, race, and median household income. A P value <0.05 was used to determine the statistical significance of the test result. All statistical analyses were done using a Statistical Package for the Social Sciences (SPSS) version 26.0 (IBM Corp., Armonk, NY).

## Results

Alcohol use disorder was most prevalent (82.2%), followed by stimulants (5.6%), cannabis (4.9%), barbiturate (4.2%), and opioid (3.1%). Alcohol use disorder was seen in a higher proportion of old-age adults (60-79 years, 68.6%), male (76.7%), and white (81.1%), and equally across all four-percentile median household income groups. Similarly, opioid and stimulant use disorders were found to be highest in old-age adults (60-79 years, 66.7% and 55.6% respectively), male (66.7% and 90% respectively), and white (83.3% and 72.2% respectively), but more amongst those with median household income above 50th percentile. Cannabis use disorder was prevalent in middle-aged adults, 40-59 years (67.4%), male (55.6%), and white (86.1%). PD inpatients with cannabis use disorder were highest amongst those having the lowest income of 0-25th percentile (50.0%). On the contrary, barbiturate use disorder was highly evident amongst older-age adults 80 years and above (62.5%), male (62.5%), and white (75.0%). The demographic distribution of PD inpatients by spectrum of SUD is shown in Table 1.

**Table 1:** Characteristics of Parkinson’s disease inpatients by substance use disorders. SNF/INF: skilled or intermediate nursing facility.

Variable	Alcohol	Cannabis	Opioid	Stimulants	Barbiturate	P value
Number of inpatients	789	46	30	54	40	-
Age at admission, in years (%)						
40-59	18.6	67.4	16.7	44.4	0	<0.001
60-79	68.6	32.6	66.7	55.6	37.5	
above 80	12.8	0	16.7	0	62.5	
Sex (%)						
Male	76.7	55.6	66.7	90.7	62.5	<0.001
Female	23.3	44.4	33.3	9.3	37.5	
Race (%)						
White	81.1	86.1	83.3	72.2	75.0	<0.001
Black	7.7	0	16.7	9.3	0	
Hispanic	7.9	0	0	9.3	12.5	
Other	3.3	13.9	0	9.3	12.5	
Median household income, in percentile (%)						
0-25^th^	27.4	50.0	33.3	27.3	37.5	<0.001
26^th^-50^th^	28.1	25.0	0	27.3	12.5	
51^st^-75^th^	23.8	25.0	16.7	36.4	12.5	
76^th^-100^th^	20.7	0	50.0	9.1	37.5	
Severity of illness (%)						
Minor loss of function	22.2	43.5	0	9.3	12.5	<0.001
Moderate loss of function	50.2	56.5	50.0	64.8	37.5	
Major loss of function	27.6	0	50.0	25.9	50.0	
Disposition						
SNF/INF	53.4	11.1	33.3	61.1	87.5	<0.001

The severity of illness is statistically higher in PD inpatients with comorbid opioid and barbiturate use disorders with major loss of body function, closely seconded by alcohol and stimulant use disorder cohorts (27.6% and 25.9%, respectively). Loss of body functioning in PD inpatients increases with advancing age as elders (80 years and above) have six-fold increased odds (95% CI 5.32-6.37) compared to 40-59 years. Males have marginally higher odds (OR 1.2, 95% CI 1.12-1.22) than females, and blacks have higher odds (OR 1.7, 95% CI 1.56 - 1.81) than whites for major loss of functioning. Among the SUD, opioid use disorder has the highest odds of association with major loss of functioning in PD inpatients by 3.8-fold (95% CI 1.96-7.35) followed by barbiturate use disorder (OR 2.3, 95% CI 1.23-4.41) and stimulant use disorder (OR 1.9, 95% CI 1.07-3.38) as shown in Table 2.

**Table 2:** Odds of association for major loss of functioning in Parkinson’s disease inpatients.

Variable	Odds ratio	95% confidence interval		P-value
		Lower	Upper	
Age at admission, in years				
40-59	Reference			
60-79	2.68	2.46	2.93	<0.001
above 80	5.82	5.32	6.37	<0.001
Sex				
Male	1.16	1.12	1.22	<0.001
Female	Reference			
Race				
White	Reference			
Black	1.68	1.56	1.81	<0.001
Hispanic	1.06	0.97	1.12	0.191
Other	1.02	0.93	1.11	0.708
Median household income, in percentile				
0-25^th^	Reference			
26^th^-50^th^	0.97	0.92	1.03	0.340
51^st^-75^th^	0.99	0.94	1.05	0.802
76^th^-100^th^	1.03	0.97	1.09	0.372
Comorbid substance use disorders				
None	Reference			
Alcohol	1.57	1.32	1.86	<0.001
Cannabis	<0.001	<0.001	-	0.997
Opioid	3.79	1.96	7.35	<0.001
Stimulants	1.90	1.07	3.38	0.029
Barbiturate	2.33	1.23	4.41	0.009

During inpatient management for PD, the LOS varies broadly with comorbid SUD. PD inpatients with barbiturate use disorder had the higher mean LOS and total charges of 17.4 days and $68,922, respectively, whereas it was lowest in PD inpatients with opioid use disorder (2.8 days and $23,311). Elders (80 years and above) have eight-fold increased odds (95% CI 7.39-8.49) compared to 40-59 years, and blacks have a 1.5-fold higher odds (95% CI 1.39-1.60) than white for disposition to SNF/INF. PD inpatients with barbiturate use disorder have six-fold increased likelihood (95% CI 2.33-15.67) of disposition to SNF/INF, followed by PD inpatient with stimulant (OR 2.6, 95% CI 1.57-4.39) and alcohol (OR 2.3, 95% CI 1.97-2.68) use disorders as shown in Table 3.

**Table 3:** Odds of association for disposition to nursing facilities in Parkinson’s disease inpatients.

Variable	Odds ratio	95% confidence interval		P-value
		Lower	Upper	
Age at admission, in years				
40-59	Reference			
60-79	2.95	2.76	3.15	<0.001
Above 80	7.92	7.39	8.49	<0.001
Sex				
Male	0.89	0.87	0.93	<0.001
Female	Reference			
Race				
White	Reference			
Black	1.49	1.39	1.60	<0.001
Hispanic	0.77	0.71	0.83	<0.001
Other	0.78	0.72	0.84	<0.001
Median household income, in percentile				
0-25^th^	Reference			
26^th^-50^th^	0.92	0.87	0.97	0.001
51^st^-75^th^	0.92	0.88	0.97	0.001
76^th^-100^th^	0.85	0.81	0.89	<0.001
Comorbid substance use disorders				
None	Reference			
Alcohol	2.29	1.97	2.68	<0.001
Cannabis	0.88	0.46	1.69	0.702
Opioid	0.62	0.32	1.22	0.168
Stimulants	2.62	1.57	4.39	<0.001
Barbiturate	6.04	2.33	15.67	<0.001

## Discussion

Our study aimed to discern the differences in demographics and DBS utilization patterns among PD inpatients with SUD and evaluate the impact of the spectrum of SUD on functionality and discharge disposition among hospitalized patients with PD. We found that alcohol use disorder was most prevalent (82.2%) and opioid use disorder being the least prevalent. We found that blacks, males and advanced age, and SUD (except cannabis use) were significantly associated with higher odds of major loss of functioning in patients with PD. Furthermore, advanced age, blacks, and females were associated with a higher odd for discharge to SNF/INF.

A higher proportion of male patients with PD had comorbid SUD, with a male: female ratio highest for stimulants (9:1), alcohol (4:1), and opioid and barbiturate (2:1). In general, SUD are more common in males than females due to biological and environmental predispositions [8, 9]. Previous studies also found that motor dysfunction is correlated with increased use of alcohol, opioid, and stimulants, and is associated with a functional deficit of the nigrostriatal system leading to increased disease severity in patients with PD [10,11]. The prevalence of SUD varies with gender, and so is the impact on disease severity and disposition of PD patients. Male patients with PD are more likely to have major loss of functioning by 16% than females, which is supported by existing literature as females have better improvements in mobility and cognition [12-14].

When a higher proportion of barbiturate use disorder was seen in the elderly population (above 80), then other SUD were prevalent in older adults (60-79 years), and two-third of the PD patients with cannabis use disorder were middle age (40-59 years). Furthermore, we found a strong association between older age (above 80 years) and major loss of functioning (increases by six times). These findings are consistent with results from past studies, which show that advanced age remains a significant risk factor for increased severity of PD even after adjusting for disease duration [12,15]. PD is a progressive neurodegenerative disease, and as neuronal loss progresses, disease severity is expected to worsen with age. We also found a strong association between advancing age and a higher likelihood of disposition to nursing facilities. Sharma et al. found that the odds for adverse disposition requiring institutionalized care were more likely in elderly PD patients (>65 years) [16]. 

Black patients with PD were 68% more likely to have major loss of functioning compared to whites. Prior research has evaluated racial disparities in outcomes for PD patients. Hemmings et al. reported greater severity of parkinsonian signs, symptoms, and disability in blacks with PD compared to whites. They also noted significant racial differences in the management of PD as blacks were less likely to receive antiparkinsonian medications and less likely to receive the newer medications on the market. Other possible factors include differences in accessing healthcare and socioeconomic status [17]. Dahodwala et al. also reported that although blacks were less likely to be diagnosed with PD, they were four times less likely to receive PD treatment than whites [18]. We found that blacks were 49% more likely to require discharge to SNF/INF than whites. While there is no prior research on racial differences in discharge disposition among PD patients, one possible reason for this is the higher likelihood of major PD severity among blacks observed in our study.

Regarding SUD among PD patients, we observed that alcohol use disorder was the most prevalent (82%) and was associated with 1.6 times increased odds for major loss of functioning and adverse disposition to SNF/INF by two times. One explanation for this is the likelihood of worsened PD due to alcohol-induced oxidative stress leading to neurotoxic brain damage [19,20]. Another potential reason could be the finding of depleted nigrostriatal monoaminergic terminals in the brains of chronic alcoholics with or without loss of neurons from the substantia nigra [21]. While our results do not imply causality, it adds to the existing literature and should prompt further research regarding the relationship of alcohol use disorder and PD and its impact on patient outcomes.

Furthermore, our data analysis shows that except for cannabis use disorder, all other comorbid SUD were associated with significantly higher odds for major loss of functioning among PD patients. The reason for this lower odd of association with cannabis use remains unclear. Cannabis may show promise in PD as a form of medical marijuana. Current evidence is limited to animal studies which show that cannabis possesses neuroprotective properties against the progressive degeneration of nigrostriatal dopaminergic neurons occurring in PD and may attenuate some of the motor symptoms associated with PD. However, further randomized clinical trials and longitudinal studies are required to determine the long-term benefits and consequences of cannabis use in PD patients [22].

We also found significant relationships between SUD and PD severity. Comorbid opioid use disorder was associated with almost four-fold higher odds for major loss of functioning. The neurobiochemical derangements in PD compromise multiple neurochemical substrates, including dopamine, norepinephrine, serotonin, acetylcholine, and glutamate systems, resulting in several motor impairments. Currently, therapy is focused on dopamine depletion, which also brings about additional motor dysfunction, including dyskinesias [23,24]. Delta opioid receptors are densely located in the basal ganglia, and studies, although inconclusive, have shown that activating these receptors leads to motor improvements, especially for dyskinesias resulting from the use of l-dopa [25,26].

Overall, these findings suggest that comorbid SUD among PD inpatients is associated with more severe illness and prolonged care. This may result in higher medical care costs for PD patients and their caregivers. PD is a progressive degenerative disorder that is associated with a significant economic burden for both patients and caregivers [27,28]. Due to the complex and multifaceted nature of PD, a wide range of health professionals are involved in care. Prior research and treatment guidelines have stressed the benefits of a collaborative care model that involves a team of neurologists, psychiatrists, nurses, speech therapists, physiotherapists, social workers, and occupational therapists [29,30].

Our study has some limitations. First, being a cross-sectional study, our results only imply association and cannot offer any information on causality between PD and SUD. Also, utilization of inpatient administrative data (and not patient-level data) as the unit of analysis limits the generalizability for all patients with PD. Therefore, it is recommended that future research utilize clinical data or longitudinal data to evaluate the relationship between SUD and PD.

## Conclusions

SUD are prevalent among PD patients and are associated with more severe illness with body loss of functioning and prolonged inpatient care with adverse disposition to nursing facilities. Therefore, healthcare providers should screen for and treat SUD among this at-risk population to lessen the disease burden and potentially reduce costs of care. A multidisciplinary care model including collaborative psychiatric and addiction management is required to manage SUD among PD patients to lessen disease severity, slow down the disease progression and improve the patient's quality of life.
